# S-duct flow distortion with non-uniform inlet conditions

**DOI:** 10.1177/09544100221101669

**Published:** 2022-05-24

**Authors:** Matteo Migliorini, Pavlos K Zachos, David G MacManus, Pierre Haladuda

**Affiliations:** Centre for Propulsion and Thermal Power Engineering, School of Aerospace Transport and Manufacturing, 2717 Cranfield University, Cranfield, Bedfordshire, UK

**Keywords:** Intake, S-duct, flow distortion, peak distortion, extreme value theory, inlet conditions

## Abstract

Convoluted aero-engine intakes are often required to enable closer integration between engine and airframe. Although the majority of previous research focused on the distortion of S-duct intakes with undistorted inlet conditions, there is a need to investigate the impact of more challenging inlet conditions at which the intake duct is expected to operate. The impact of inlet vortices and total pressure profiles on the inherent unsteady flow distortion of an S-duct intake was assessed with stereo particle image velocimetry. Inlet vortices disrupted the characteristic flow switching mode but had a modest impact on the peak levels and unsteady fluctuations. Non-uniform inlet total pressure profiles increased the peak swirl intensity and its unsteadiness. The frequency of swirl angle fluctuations was sensitive to the azimuthal orientation of the non-uniform total pressure distribution. The modelling of peak distortion with the extreme value theory revealed that although for some inlet configurations the measured peak swirl intensity was similar, the growth rate of the peak values beyond the experimental observations was substantially different and it was related with the measured flow unsteadiness. This highlights the need of unsteady swirl distortion measurements and the use of statistical models to assess the time-invariant peak distortion levels. Overall, the work shows it is vital to include the effect of the inlet flow conditions as it substantially alters the characteristics of the complex intake flow distortion.

## Introduction

The aviation community is constantly striving for more efficient, reliable, environmental-friendly and sustainable air transport solutions which are aligned with future societal and market needs.^1^ While the research on conventional aircraft design optimization may be reaching development maturity with focus on incremental enhancement, interest in novel aircraft configurations is emerging. In some of these novel architectures, the propulsion system is closely coupled to the aircraft fuselage to reduce frontal area, wakes and to take advantage from boundary layer ingestion.^2^ Recent feasibility studies^3–5^ predicted a reduction of fuel consumption up to 5% in comparison with UHBR engines.^6^ The benefit of fuel economy can be even higher if this technology is used in conjunction with blended wing body aircraft design, which could save more than 20% in fuel burn per seat mile.^6^ In some configurations, the engine is embedded or semi-embedded in the fuselage and the air is often ducted into convoluted intakes which feed the propulsion system.^2^ The flow associated with these intakes is highly unsteady and distorted due to the flow separations and the secondary flows promoted by the duct bends.^7–9^ The flow distortion has a direct impact on the propulsion system performance and reliability,^10,11^ since it can penalize the aerodynamic stability^12,13^ and can produce forced excitations which can adversely affect the mechanical system compatibility.^14,15^ Moreover, it has been demonstrated that inlet entry conditions have an impact on the S-duct aerodynamics.^16,17^ Often, experimental testing and S-duct CFD optimization are required to assess the operability of the propulsion system for a range of different inlet configurations.^18–20^

Among the non-uniform inlet conditions, the ingestion of vortices and total pressure profiles are of interest especially for the new generation of aircraft. The ingestion of vortices in aero-engines has been investigated for the characterization of ground vortices.^21^ This topic is still of importance for aircraft manufacturers, especially in relation to the ingestion of foreign object debris and for cross-wind conditions.^22,21^ Depending on the configuration, the closer integration of the engine with the fuselage may promote the formation of vortices which arise from the aerodynamic surfaces. In addition, the engine may be exposed to vortices which originate from the inner wing leading edge, canards, strakes and forebody.^23^ Understanding the interaction of the vortices with the complex flow field of S-duct intakes is believed to be a cornerstone for the successful integration of the complex intakes with the propulsion system.^24^ In addition, the use of devices to assess the impact of inlet total pressure distortion is an established approach.^25^ Many design methods and analytical solutions have been used throughout the years^26^ and, more recently, additive manufacturing enabled the production of gauzes to generate non-uniform total pressure profiles to a thick approaching boundary layer.^3^ The integration of CFD and experimental design methods demonstrate that is possible to generate swirl, total pressure, or combined swirl and total pressure distortion.^27–29^

Recent advanced experimental methods have provided a notable advance in the characterization of the flow distortion in S-duct intakes. Stereo particle image velocimetry (S-PIV) has been applied to assess the unsteady 3D velocity and swirl distributions at the aerodynamic interface plane (AIP). This offered synchronous non-intrusive measurements with a data density up to two orders of magnitude greater than traditional measurement techniques.^30,31^ S-duct with a relatively high centreline offset generally presented greater unsteady swirl distortion levels.^32^ Proper orthogonal decomposition (POD) revealed the fundamental coherent structures which drive the unsteady swirl patterns at the AIP.^33^ The extreme value theory (EVT) estimated upper bounds of the swirl fluctuations beyond the measured dataset.^34^ It was demonstrated as a tool to predict the peak distortion for events whose observations would require prohibitively long testing duration.^35^ The introduction of time-resolved particle image velocimetry (TR-PIV) enabled the assessment also of the characteristic spectral signature of the swirl distortion.^36^ These fluctuations were found to fall in the range in which disturbances may trigger instability of the compressor system, which is from 1-per-rev to the passing frequency of the fan blades for axial compressors.^37^

However, there is little evidence of the impact of non-uniform inlet conditions on the inherent flow distortion of S-duct intakes. Only a few published studies have assessed the influence of inlet vortices for S-duct intakes. Most of the research focused on the use of inlet vortex generators as flow control devices to reduce the separations within the intake, the strength of the secondary flows and, consequently the engine fan face distortion.^38–40^ For example, a study on the reduction of the unsteadiness of the peak swirl with sub-boundary layer vortex generators was conducted by Tanguy et al.^41^ Research focused also on methods to generate vortices for engine inlet applications and on the vortex development from the source (i.e. ground vortex or wingtip vortex) to the intake.^42–44^ A few experimental studies focused on the impact of inlet vortices onto the S-duct aerodynamics. Wendt and Reichert^45^ demonstrated that the inlet vortex position influenced the swirl and pressure distortion at the S-duct outlet, but the findings were limited by the experimental capability.^45^ Similar conclusions were drawn by Mitchell^46^ who found that the wing tip vortex trajectory and impact on compressor surge margin depended on the vortex position along the main vertical intake axis. The greater impact on surge margin was found for the ingestion of the vortex at the centre of the intake, which reduced the non-dimensional surge margin by up to 6%. The inlet vortex had a more significant impact at higher compressor rotational speeds. It also revealed that the rotation direction of the vortex plays a significant role on the impact on compressor surge margin. For a vortex rotating counter to the engine, the surge margin reduced by up to 6%, while for a co-rotating vortex the surge margin reduced by 3%.^46^ More recent computational studies by Mehdi^47^ confirmed these observations. Contra-rotating vortices drove a raise in compressor pressure ratio, and at the same time, a reduction of isentropic efficiency compared with the effect of co-rotating ones. The greatest loss in compressor pressure ratio was found for high-strength vortices ingested near the hub. He also demonstrated that for constant total vortex circulation, vortices with smaller core size were more detrimental to compressor performance than larger ones because they caused larger separations on the rotor blades.^47^ However, in general, there is a lack of investigation of the impact of inlet vortices on the unsteady flow distortion characteristics of S-duct intakes.

Similarly, while a considerable amount of research focused on generalized inlet pressure distortion, very few studies have been published on the impact of boundary layer ingestion on S-duct unsteady aerodynamics. Rein and Koch^48^ demonstrated that the distortion at the AIP increases proportionally with the thickness of the inlet boundary layer. Thick boundary layers were found to reduce also the pressure recovery on the intake^49–51^ and to promote non-uniform radial pressure loading on the compressor blades which could trigger stall inception.^4^ Different azimuthal orientations of the approaching boundary layer were also investigated for intakes under yaw and pitch angles^52,53,18^ were found to notably influence the fan-face pressure and swirl distortion. However, the associated peak distortion levels were not assessed as a part of these previous studies and most studies were limited to investigations on the time-averaged components.^50^ The understanding of peak distortion levels is a key aspect for the evaluation of the engine response to the inlet distortion. Indeed, it is believed that peak fluctuations of pressure, vorticity and velocity can all play a role in the spike-type stall inception mechanism, which is a source of instability for modern, highly loaded compressors.^54^ Moreover, most of the intake distortion work is based on canonical configurations with uniform conditions, and there is little information on the impact of inlet conditions on the inherent distortion of the S-duct intake.

Within this context, the aim of this work is to quantify the impact of non-uniform inlet conditions, total pressure profile and inlet vortices on the unsteady aerodynamics of the intake, and to characterize the peak level of distortion with a statistical modelling of the extreme events. Flow-conditioning devices are introduced at the inlet of an S-duct intake to generate non-uniform inlet conditions including vortices of different strength and position, and total pressure profiles with different thickness and azimuthal orientation with respect to the intake. The effect of these non-uniform inlet conditions on the inherent S-duct flow distortion is assessed with S-PIV measurement at the AIP. Flow distortion metrics and unsteady analysis methods are applied to assess the measured peak distortion levels for the various test cases, and the extreme value theory is applied as a statistical model to estimate the upper bounds of the unsteady distortion distribution for observations beyond the experimental testing time.

## Experimental details and analysis methods

### Test rig for S-duct intakes

**Figure 1.**
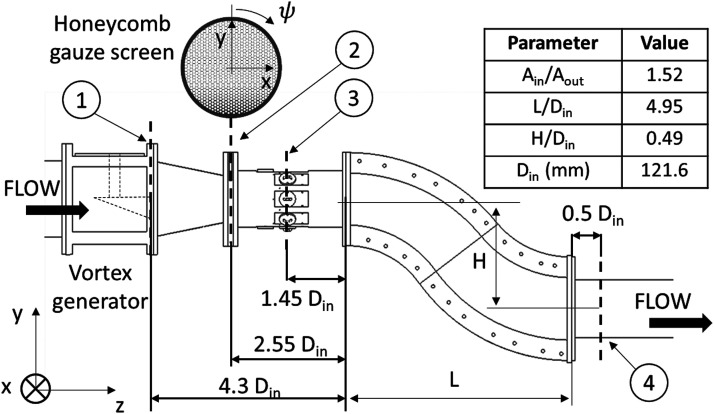
Cranfield test facility schematic: 1 – Trailing edge of the vortex generator, 2 – position of the honeycomb gauze screen, 3 – PIV measurement plane at S-duct inlet and 4 – PIV measurement plane at S-duct AIP.

A comprehensive description of the complex intake facility is outlined in Zachos et al.^30^ It comprises a diffusing S-duct intake similar to that of Garnier et al.^9^ The rig operating point was set at Mach 0.27, and it was measured at the inlet plane located 1.45D_in_ upstream of the inlet of the S-duct intake (plane 3, Figure 1).

#### Inlet vortices

**Figure 2.**
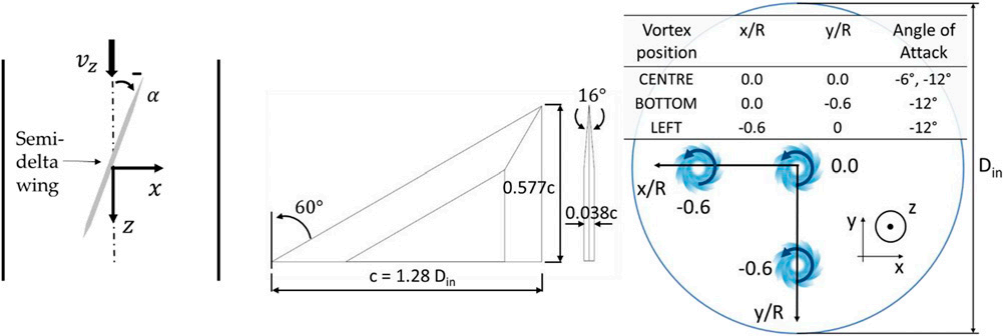
Semi-span delta wing dimensions and definition of the angle of attack (AoA) relative to the incoming flow (top view).

Vortices at the inlet of the S-duct were generated with a semi-span delta wing^55,56^ which was positioned 4.3D_in_ upstream of the S-duct inlet plane (about 3.35 chords). It had a maximum chord length c = 1.28 D_in_, a span of 0.577 c, a chamfer half-angle of 8° at the edges, a sweep angle of 60° and a maximum thickness of 0.038 c. The half-delta wing was supported with a NACA 0012 wing section strut. A splitter disk with sharp edges was placed between the strut and the delta wing to reduce the generation of spurious vortices in the boundary between strut and delta wing. The vortex size and circulation has been controlled by changing the angle of attack (AoA) (Figure 2). The effect of the vortex size and strength was assessed using an AoA = −6° and −12°. Given the negative incidence of the delta wing, the vortex rotates anti-clockwise when viewed from downstream. The delta wing was also translated along the vertical (y) and lateral (x) axes to assess the influence of the vortex position on the AIP flow distortion.

The vortex characteristics have been assessed with TR-PIV measurements at the S-duct inlet plane (Figure 1). The vortex core size and core radius^57^ were identified with the vorticity disk method.^58^ The vortex position was assessed with a conditional average of the vortex over 10,000 TR-PIV snapshots. Only the negative vorticity was considered to compute the vortex circulation in order to focus on the vorticity generated by the delta wing only. A domain of r/D_in_ = 0.20 and a resolution of 0.12 r_core_ were sufficient to capture 98% of the total vortex circulation. The measured vortex characteristics for the different inlet positions are reported in Table 1.

**Table 1.** Measured inlet vortex characteristics at 1.45 D_in_ upstream the S-duct intake.

x/R	y/R	AoA	$r_{\mathrm{core}}/D_{\mathrm{in}}$	$\;\Gamma_{0}/\left(\bar{v_{z}}D_{in}\right)$
−0.01	−0.09	−6°	0.03	−0.022
0.04	0.00	−12°	0.04	−0.151
0.12	−0.65	−12°	0.04	−0.158
−0.54	−0.05	−12°	0.04	−0.184

#### Inlet total pressure distributions

For the generation of non-uniform total pressure distributions at the inlet of the S-duct, distortion gauzes were placed at plane 2 (Figure 1), 2.55D_in_ upstream of the S-duct. A honeycomb gauze screen with variable porosity^3^ reproduced a boundary layer–type total pressure profile^59^ whose thickness was δ/D_in_ = 0.332 (Profile A)^4^ and $Re_{\delta }$ = 6.3 × 10^4^, which is substantially thicker than the nominal profile with δ/D_in_ = 0.04 and $Re_{\delta }$ = 4.6 × 10^3^. Profile A may represent a nominal boundary layer which develops in full-scale blended wing body (BWB) of about $\delta /D_{in}$ = 0.30.^48^ An additional total pressure profile (Profile B) with δ/D_in_ = 0.572 was also included in this study to investigate the impact of profiles with a different thickness and to simulate the approaching conditions of a thick boundary layer under higher angles of attack. The baseline configuration of the profiles presented the main total pressure loss region in the lower part of the inlet. These could also rotate azimuthally by ψ= 90° to simulate an operating condition of the intake at angles of pitch, yaw or cross-wind.^52^

#### TR-PIV experimental setup

Time-resolved PIV was used for the measurements of the velocities at plane 4 (AIP, 0.4D_out_, Figure 1). Di-ethyl-hexyl sebacate particles of approximately 1 μm seeded the flow and were spotlighted with a pulsed Nd:YAG laser on cross-flow planes 3 and 4 (Figure 1). A pair of CMOS cameras at each side of the rig were used to record the PIV images. The cameras had 16,600 fps as maximum frame rate, and the sensor resolution was 1280 × 800 px^2^ (1MP). The acquisition frequency was 4 kHz, which is substantially higher than the main flow frequency of around St = 1.0.^36^ For each case, 20,000 instantaneous velocity snapshots were acquired to ensure that the streamwise velocity component and its standard deviation converged to 0.2% and 0.4%, respectively.^36^ The velocity results had a spatial resolution of 0.0153D_out_ across the AIP. In the current analysis, the data was considered only within $r<0.95R_{AIP}$ to ensure that no spurious vectors caused by laser light reflections near the domain boundaries influenced the measurements. The measurement grid counted approximately 2900 velocity vectors across the AIP. A conventional flow distortion measurement system would usually provide 40 total pressure measurements across the plane^9^; therefore, the number of measurements is more than two orders of magnitude greater for PIV compared to conventional measurements techniques. A disparity correction was used to limit the bias errors caused by the relative position of the calibration plate and the laser light. The velocity components uncertainty was assessed with the method by Raffel et al.^60^ which is arguably the most widely used method in the literature among others.^61,62^ This yielded an uncertainty on the velocity components equal to 3.3% of the area-averaged of the time-averaged streamwise velocity at the AIP. Based on the propagation of the velocity uncertainties onto the derived metrics, the uncertainty on the swirl angle (α), ring-based swirl intensity (SI) and on the inlet vortex circulation (Γ) is estimated at 1.7°, 0.20° and 13%, respectively. The velocity measurements are normalized against the area-averaged of the time-averaged streamwise velocity $\left(\bar{v_{z}}\right)$ measured with uniform inlet conditions (δ/D_in_ = 0.04) with no inlet vortices.

#### Methods for the flow distortion assessment

**Figure 3.**
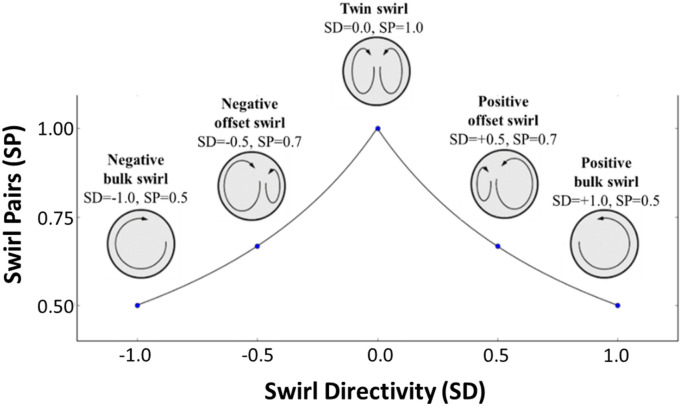
Correlation between swirl patterns and SAE descriptors adapted from Ref. 28.

The swirl distortion patterns have been evaluated with the SAE industrial distortion descriptors.^28^ The swirl angle at the AIP is positive in the counter-clockwise direction considering the right-hand rule and the out-of-plane velocity vector. The swirl distortion is evaluated with swirl descriptors computed based on a polar grid of rings at the AIP. The swirl intensity (SI) measures the potency of the swirl, the swirl directivity (SD) the main sense of rotation and the swirl pair (SP) the number of contra-rotating vortices pairs. A more detailed description is reported by Zachos et al.^30^ and an overview of the main swirl patterns is shown in Figure 3. The Delaunay triangulation method is used to interpolate the TR-PIV data at the radial locations.^63^ The swirl descriptors are evaluated at each timestep of the PIV acquisition. To depict the relative probability of the swirl patterns, the analysis adopted the joint-probability density functions (j-PDF) introduced by Gil-Prieto et al.^36^ The probability to detect a certain swirl pattern is computed through the integration of the PDF on a discretization of the SI-SP grid (equation (7)) with a resolution of 0.03 for both descriptors. The analysis focuses on the swirl descriptors at a radial location close to the compressor blade tip (r/R = 0.84) since this is the region where stall inception is believed to originate from^54^

(1) $$P\left(SD_{A}\leq SD\leq SD_{B},\;SP_{A}\leq SP\leq SP_{B}\right)=\int_{SP_{A}}^{SP_{B}}\int_{SD_{A}}^{SD_{B}}PDF\;dSD\;dSP$$

For analysing the spectral signature of the swirl distortion, an average periodogram method was introduced.^64^ Each PIV dataset has been divided into 20 parts. The frequency leakage was limited with the application of a Hann window. The resolution of the measured frequency is ΔSt  = 0.01 approximately and frequency contributions up to St  = 5.0 could be identified. The contribution of each frequency band normalized by the overall area-averaged variance has been computed to identify the highest contribution across each frequency band.

#### Modelling of the extreme distortion events

The extreme value theory (EVT) was applied to the unsteady data to estimate the peak distortion levels for observations beyond the experimental testing time. The EVT method was introduced in the context of the prediction of peak flow distortion by Jacocks et al.^65^ and it has also been used in previous work by Gil-Prieto et al.^34^ and Tanguy et al.^35^ to predict the peak total pressure and peak swirl distortion, respectively, in the context of S-duct flow distortion. This work applies the EVT in the peak-over-threshold formulation which is based on the threshold models reported by Coles,^66^ which considers events as extreme if they exceed a certain threshold μ. For a large enough threshold μ and number of observations n, the limit distribution of the k excesses $Y_{i}$ (equation (2)) tends to be a generalized Pareto distribution (equation (3)). In this model, the shape parameter is ξ and the scale parameter is σ. The distribution of the k excesses over the threshold is limited at the upper bound at $U_{b}=\mu -\left(\sigma /\xi \right)$ for the case where the shape factor is ξ<0.  These parameters can be estimated through the maximization of the logarithmic likelihood function (equation (4)).^66^ The shape and scale parameters (ξ and σ, respectively) are used to estimate the probability of an extreme value $x_{m}$ exceeding the threshold $\zeta_{u}=k/n$ on average once every m observation (equation (5)). The error of the extreme value $x_{m}$ is found by applying the delta method (equation (6)), considering the gradient $\nabla x_{m}$ (equation (7)) and the matrix of the variance-covariance VC (equation (8)). The matrix VC collects the variance errors of the parameters $\zeta_{u}$, σ and ξ whose variance is obtained from the analytical solutions of the model parameters (equations (9)–(12)).^67^ Finally, the 95% confidence interval CI for the model predictions can be derived by assuming that $x_{m}$ follows a normal distribution and a quantile $z_{\alpha /2}=1.96$ (equation (13))

(2) $$\;\;Y_{i}=\left(X_{i}-\mu \right)|X_{i}>\mu$$

(3) $$H\left(y\right)=P\left\{Y\leq y\right\}=1-{\left(1+\frac{\xi y}{\sigma }\right)}^{-1/\xi }$$

(4) $$l\left(\xi ,\sigma \right)=\sum_{i=1}^{k}log\left(\frac{dH}{dy}\left(Yi,\sigma ,\xi \right)\right)=-k\;log\left(\sigma \right)-\left(1+1/\xi \right)\sum_{i=1}^{k}log\left(1+\frac{\xi Y_{i}}{\sigma }\right)$$

(5) $$x_{m}=\mu +\frac{\sigma }{\xi }\left[{\left(m\zeta_{u}\right)}^{\xi }-1\right]$$

(6) $$var\left(x_{m}\right)=\nabla x_{m}^{T}VC\nabla x_{m}$$

(7) $$\nabla x_{m}^{T}=\left[\frac{\partial x_{m}}{\partial \zeta_{u}},\frac{\partial x_{m}}{\partial \sigma },\frac{\partial x_{m}}{\partial \xi }\right]$$

(8) $$VC=\left[\begin{array}{ccc} var\left(\zeta_{u}\right) & 0 & 0\\ 0 & var\left(\sigma \right) & cov\left(\xi ,\sigma \right)\\ 0 & cov\left(\xi ,\sigma \right) & var\left(\xi \right) \end{array}\right]$$

(9) $$var\left(\zeta_{u}\right)=\zeta_{u}\left(1-\zeta_{u}\right)/n$$

(10) $$var\left(\xi \;\right)={\left(1+\xi \right)}^{2}/k$$

(11) $$var\left(\sigma \right)=2\left(1+\xi \right)\sigma^{2}/k$$

(12) cov(ξ,σ)=−(1+ξ)σ/k

(13) $$CI=x_{m}\pm z_{\alpha /2}\sqrt{var\left(x_{m}\right)}$$

## Results

### Impact of inlet vortex on the AIP flow distortion

**Figure 4.**
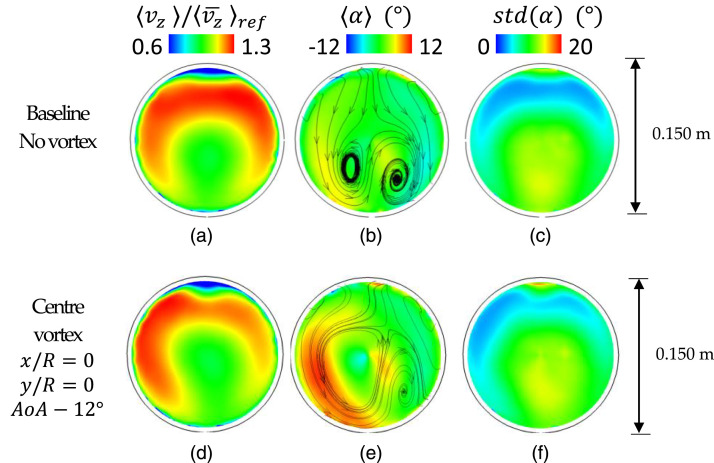
Time-averaged components at the AIP. Streamwise velocity, swirl angle and unsteadiness of the swirl angle. Conditions: uniform inlet and inlet vortex.

The characteristic flow topology for S-duct intakes is assessed with the TR-PIV measurements at the AIP. The velocity measurements are normalized against the time-averaged, area-averaged streamwise velocity $\left({\bar{v_{z}}}_{ref}\right)$ with baseline inlet conditions (δ/D_in_ = 0.04) with no inlet vortex. The time-averaged flow topology with uniform inlet flow with no vortices is in agreement with previous assessments^30,32,68^ with the same S-duct configuration (Figure 4(a)–(c)). The typical S-duct–paired swirl distribution is also visible in the time-averaged swirl angle α   (Figure 4(b)) which is mainly associated with the secondary flows which develop due to the first S-duct bend.^8^ The swirl angle varies between ±8° across the AIP. However, the unsteady swirl angle fluctuations values can be up to std(α) = 14° at the centre of the AIP (Figure 4(c)).

The presence of an inlet vortex at the centre of the inlet section promoted the development of the inherent duct secondary flows, especially with the delta wing AOA = −12° (Figure 4(d) and (f)). The left Dean vortex, which is rotating anti-clockwise as the inlet delta wing vortex, strengthens and becomes predominant, and consequently, the time-averaged maximum swirl angle increases from +8° (baseline case, Figure 4(b)) to +12° (AoA = −12°, Figure 4(e)). This effect is also visible on the out-of-plane velocity distribution for which the magnitude increases towards the left side of the domain (Figure 4(a) and (d)). This vortex increased the positive time-averaged peak swirl located in the left side of the domain from +6° to about +11° (Figure 4(e)) relative to the baseline case (Figure 4(b)). This could represent a challenging operating condition for a compressor rotor since it will likely experience a change in blade loading when passing through this region. Indeed, if the rotor is counter-rotating relative to the vortex (anti-clockwise for this configuration), its blade incidence is likely to be increased, potentially leading to separation and mechanical stress on the blade.^47^ Interestingly, on the other hand, the unsteadiness of the swirl angle remains unaffected by the presence of the inlet vortex. The swirl angle fluctuations ranged from a minimum of 3° nearby the top of the AIP to a maximum of 14° approximately near the AIP centre (Figure 4(c) and (f)) in both baseline and vortex configurations. Although the bulk flow is redistributed, the presence of the vortex does not contribute to the unsteady fluctuations of the AIP flow distortion.

When the vortex strength was reduced (AoA = −6°), the impact on the flow distortion notably weakened. The impact on the out-of-plane velocity component $v_{z}\;$ and swirl angle α  was similar but more modest. Besides a modest change in the swirl angle distribution, the effect on the AIP flow is negligible and therefore indicating that there is a possible threshold level of ingested inlet vortex size and strength that is able to affect the inherent S-duct flow distortions at a notable level. Overall, these investigations reveal the interactions of a relatively large inlet vortex and the secondary flows that develop in S-duct intakes.

**Figure 5.**
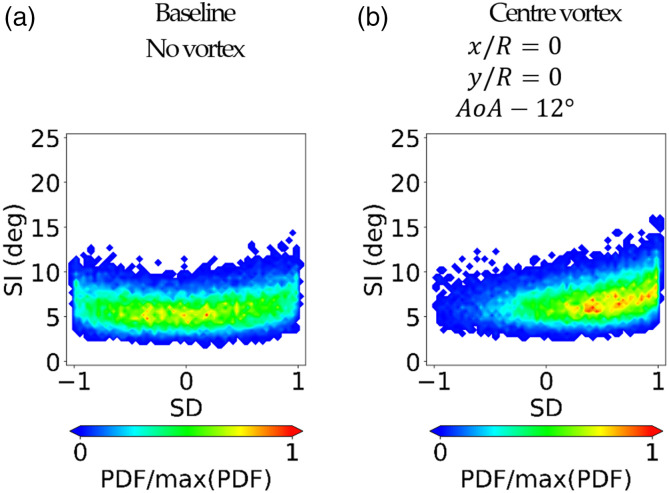
j-PDF of SI-SD distributions measured at the AIP at r/R = 0.84 for baseline and inlet vortex configuration.

The analysis of the probability of swirl patterns with uniform inlet conditions was discussed by Gil-Prieto et al.^36^ with j-PDF maps (Figure 5(a)). The alternation between these swirl patterns is caused by the swirl switching mode. Further evidence was reported in other work by MacManus et al.^31^ and Gil-Prieto and al.^68^ However, the ingestion of an inlet vortex disrupts this characteristic switching mode and promotes mainly positive bulk events (SD = +1). This is correlated with the strengthening of the anti-clockwise vortex reported in Figure 4(f). The higher the inlet vortex strength, the more evident is the predominance of positive bulk swirl events (Figure 5(b)) in contrast with the oscillatory mode of the baseline inlet conditions (Figure 5(a)). The presence of the vortex causes a slight increase of the maximum swirl intensity (SI) for bulk swirl patterns (SD = +1). In contrast to previous considerations on the time-averaged data, the disruption of the switching mode and the slight increase of SI were observed independently of the inlet vortex strength (AoA = −6° and −12°, Figure 5(b)). Thus, in summary, inlet vortices have a considerable impact on the probability characteristics of the flow topology at the AIP but have a small impact on the peak swirl intensity. Given the increase in the probability of bulk events and the previous work by Mitchell,^46^ the potential impact on a compression system will depend on the relative spinning direction of the vortex and the engine.

The evaluation of the peak distortion for the different configurations is of primary importance for the assessment of the intake-compressor compatibility and integration. It is known that the fan responds to the unsteady fluctuations of the flow^37^ and that extreme swirl distortion events can trigger instabilities for the propulsion system.^54^ As demonstrated by the j-PDF maps (Figure 5), peak swirl events are likely to be double the time-average swirl values and the statistical prediction with EVT showed even higher levels of peak distortion.^34,35^ The swirl intensity exhibited a correlation with the compressor pressure ratio and surge margin^47^; thus, the assessment of the maximum level of swirl distortion may determine the propulsion system operability limits.

### Impact of vortex ingestion position on flow distortion

**Figure 6.**
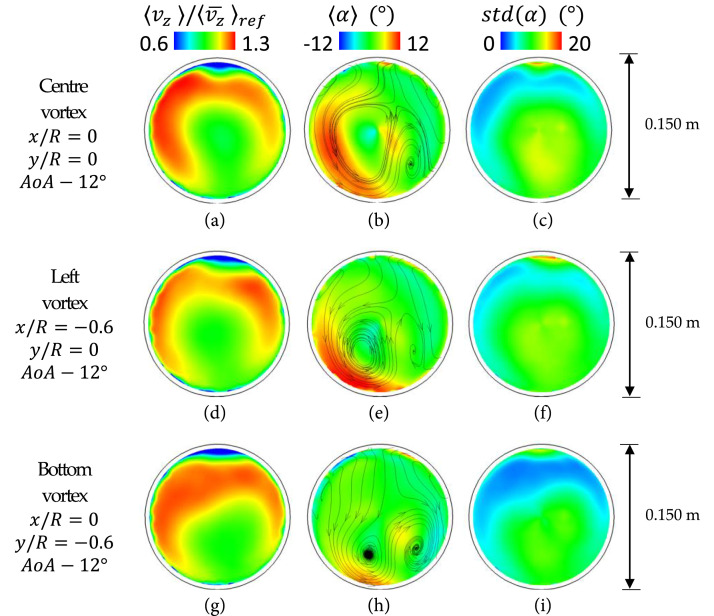
Time-averaged components at the AIP. Streamwise velocity, swirl angle and unsteadiness of the swirl angle. Conditions: inlet vortices configurations.

**Figure 7.**
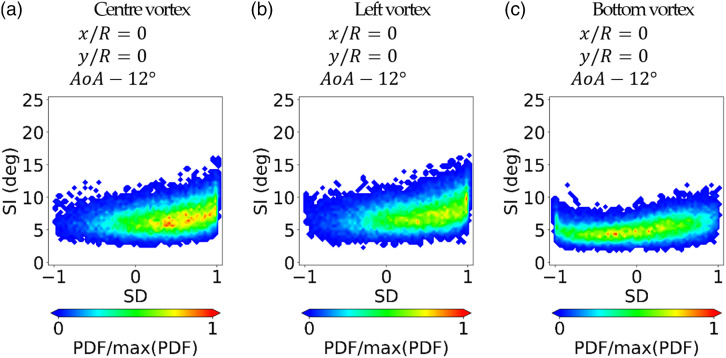
j-PDF of the SI–SD distributions measured at the AIP at r/R = 0.84 for different positions of the inlet vortex relative to the S-duct inlet.

For an embedded engine, it is possible that a discrete vortex could manifest in the forward regions of the intake system from a variety of sources, either ingested^46^ or generated within the duct.^8^ It is of interest to evaluate the interaction and the impact of additional distorted inlet conditions on the inherent distortion generated from the duct. Previous work revealed that the trajectory and the impact of inlet vortices depended on the ingestion position along the vertical intake axis.^46^ The location at which the vortex is ingested depends on many factors, such as the origin position, the direction of the flow relative to the intake and the ground clearance.^44^ In this work, the vortex generator has been translated along the vertical and horizontal direction in order to depict the influence of the vortex position relative to the inlet of the S-duct on the flow distortion at the S-duct AIP. During this process, the angle of attack of the delta wing was kept constant at AoA = −12° so that the total circulation was kept approximately constant at $\Gamma_{0}/\left(\bar{v_{z}}{\text{D}}_{\text{in}}\right)$ = −0.15 and core radius r/D_in_ = 0.04. In general, the S-duct aerodynamics is sensitive to the vortex position (Figure 6). The horizontal translation of the vortex towards the left boundary (x/R = −0.6, y/R = 0) produced a shift of the positive swirl area towards the AIP lower-left region. In this configuration (Figure 6(e)), the peak time-averaged swirl angle remains unchanged when compared to the centre vortex case (Figure 6(b)). The swirl angle unsteadiness (Figure 6(f)) is similar to the configuration of the vortex ingestion at the centre of the inlet section (Figure 6(c)). With this configuration, the unsteady swirl distribution (Figure 7(b)) exhibits a tendency towards positive bulk swirl (SD = 1) which disrupts the switching mode, and it is broadly similar to the case in which the vortex is ingested at the centre of the inlet section (Figure 7(a)). Also the peak swirl intensity (Figure 7(b)) remains the same compared to the centre vortex case (Figure 7(a)). Therefore, overall, the ingestion position at the inlet centre and left side had broadly the same impact on the AIP flow distortion.

On the other hand, the vortex translation towards the lower part of the S-duct intake (x/R = 0, y/R = −0.6) caused a more pronounced impact on the AIP flow distortion. With the vortex at the lower part of the inlet section, the out-of-plane velocity (Figure 6(g)) redistributes and becomes more uniform and more similar to the distribution of the baseline inlet configuration (Figure 4(a)). This is accompanied with a reduction of the peak swirl angle from +13° to +9° (Figure 6(h)) when compared to the centre vortex configuration (Figure 6(b)). The area-averaged swirl angle unsteadiness (Figure 6(i)) also reduced by about 25% in comparison with the central vortex position (Figure 6(c)). The vortex at the inlet lower part reduced the AIP peak swirl intensity (SI) from 16.5° to about 13° compared to the centre and left inlet vortices (Figure 7(a) and (b)). A similar reduction was observed by the time-averaged swirl angle distribution whose maximum decreased by about 4° on average compared to the centre vortex case (Figure 6(g)). This reduction of peak SI can be considered beneficial as it is also lower than the baseline case with uniform inlet flow (Figure 5(a)). The reduction in SI of about 3.5° is comparable to passive flow control studies with vortex generators at the S-duct inlet plane, which reduced the peak SI by 3° to 7° depending on the vortex generator configuration.^41^ In these previous investigations, multiple vortex generators were introduced at the bottom half of the S-duct inlet to reduce the fluctuations of the swirl angle at the AIP and promote a uniform distribution of the out-of-plane velocity component. However, while for previous work the vortices were generated within sub-boundary layer scales and influenced mostly the separation after the first S-duct bend, in this work, the vortex is on a much larger scale and interacts mainly with the bulk secondary flows. In contrast, the bottom inlet vortex ingestion did not promote the bulk swirl events (Figure 7(c)). Indeed, the switching mode is still predominant in the SI–SD distribution (Figure 7(c)).

In general, the ingestion of a vortex at the centre or left side of the S-duct inlet increased the time-averaged peak swirl angle and disrupted the flow switching mode. Instead, the ingestion of the vortex towards the duct's lower side showed a beneficial impact on the AIP flow distortion since it promoted the uniformity of the out-of-plane velocity component, and it also produced a slight reduction of the swirl angle fluctuations. Overall, the swirl intensity and peak distortion is modestly sensitive to the vortex position at the S-duct inlet and, in some cases, can even be beneficial. Nevertheless, this work demonstrates how the inlet vortex interacts with the duct flow field and walls. The effect of the inlet vortex is stronger when the vortex is close to the inlet's lower side since it interacts more strongly with the secondary flows and greatly reduces the flow distortion unsteadiness.

### Impact of inlet total pressure profiles on flow distortion

**Figure 8.**
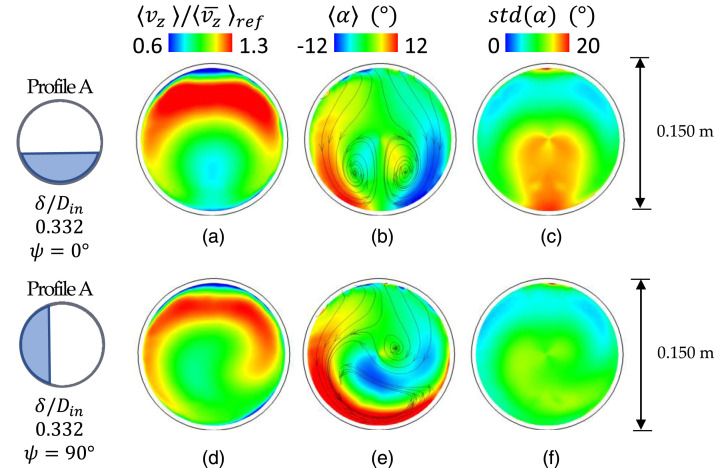
Time-averaged components at the AIP. Streamwise velocity, swirl angle and unsteadiness of the swirl angle. Conditions: inlet total pressure profile configurations.

**Figure 9.**
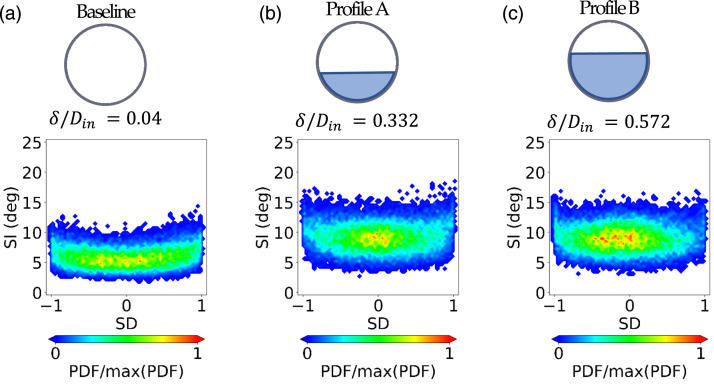
j-PDF of the SI-SD distributions measured at the AIP at r/R = 0.84 for different inlet conditions: baseline, Profile A and Profile B (δ/D_in_ = 0.04, δ/D_in_ = 0.332 and δ/D_in_ = 0.572, respectively).

The impact of inlet total pressure profiles on the flow distortion at the S-duct AIP was assessed as part of previous studies by McLelland et al.^52^ and Migliorini et al.^59^ For completeness and to enable the comparison between the influence of the inlet total pressure profiles and the impact of inlet vortices, some results by Migliorini et al.^59^ are also reported as part of the current paper. In general, the introduction of inlet total pressure profiles strengthened the secondary flows and thus produced an increase of the time-average swirl angle and its associated unsteadiness $std\left(\alpha \right)\;$ (Figure 8). The maximum swirl intensity increases from SI = 14.5° (Figure 5(a), δ/D_in_ = 0.04) to SI = 18° approximately (Figure 9(b), δ/D_in_ = 0.332 and Figure 9(c), δ/D_in_ = 0.572). However, the tri-modal swirl characteristics typical of S-duct with uniform inlets remained unchanged (δ/D_in_ = 0.04, Figure 5(a) and δ/D_in_ = 0.332, Figure 9(b)).

**Figure 10.**
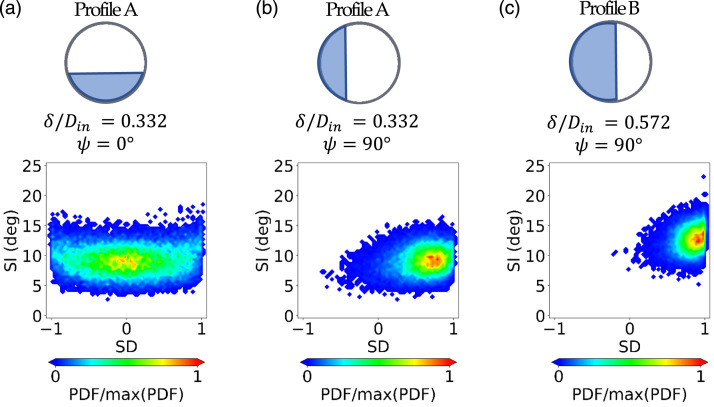
j-PDF of the SI-SD distributions measured at the AIP at r/R = 0.84 for inlet Profile A and Profile B (δ/D_in_ = 0.332 and δ/D_in_ = 0.572, respectively) at azimuthal orientations ψ=0°, 90°.

The operation of the intake at high pitch and yaw manoeuvres or cross-wind conditions was also simulated with the variation of the azimuthal orientation of the inlet total pressure profile from ψ = 0° to ψ = 90°.^52,59^ In these configurations, the strengthening of the secondary flows was polarized in one side (Figure 8(d)) and the vortex which is associated with the biased inlet condition became predominant (Figure 8(e)). Interestingly, this strengthening effect of the left Dean vortex caused by the inlet profile azimuthal rotation was similar, but more augmented, to the effect of the inlet vortex (see Section A). The bias of the swirl pattern generated a more stable condition which decreased the swirl unsteadiness. However, the impact on the peak swirl intensity depended on the profile thickness. For example, for Profile A (δ/D_in_ = 0.332) and relative to the  ψ = 0° configuration, the maximum SI reduced slightly from 18.5° to about 16.0° for ψ = 90° (Figure 10(b)) as expected due to the reduction of the swirl angle fluctuation in Figure 8(f). However, for the thickest inlet profile (Profile B, δ/D_in_ = 0.572), the maximum SI increased from 17.5° (δ/D_in_ = 0.572, ψ = 0°) to 23.5° for  ψ = 90° (Figure 10(c)). Interestingly, the flow asymmetry caused by the azimuthal rotation of the inlet profiles interacted with the secondary vortices similarly to the case of vortex ingestion. In both scenarios, the main swirl switching mode is disrupted and one of the two AIP vortices becomes predominant.

### Spectral analysis of flow distortion for different inlet conditions

The unsteady flow distortion generated by the S-duct intake could have an impact on the engine stability. For a typical propulsion system, flow disturbances in a range of frequencies between 1 engine order and the passing time of a 5-blade sector may generate instabilities in propulsion systems.^10^ This corresponds to a frequency range of Strouhal number St = 0.8–3.3 considering a representative transonic rotor.^69^ More recently, researchers showed that this range of frequencies is likely also to encompass lower frequencies which are related to the main resonant frequency of the rotor,^70^ which is in the order of St = 0.7, or disturbances of even lower frequencies.^47^

**Figure 11.**
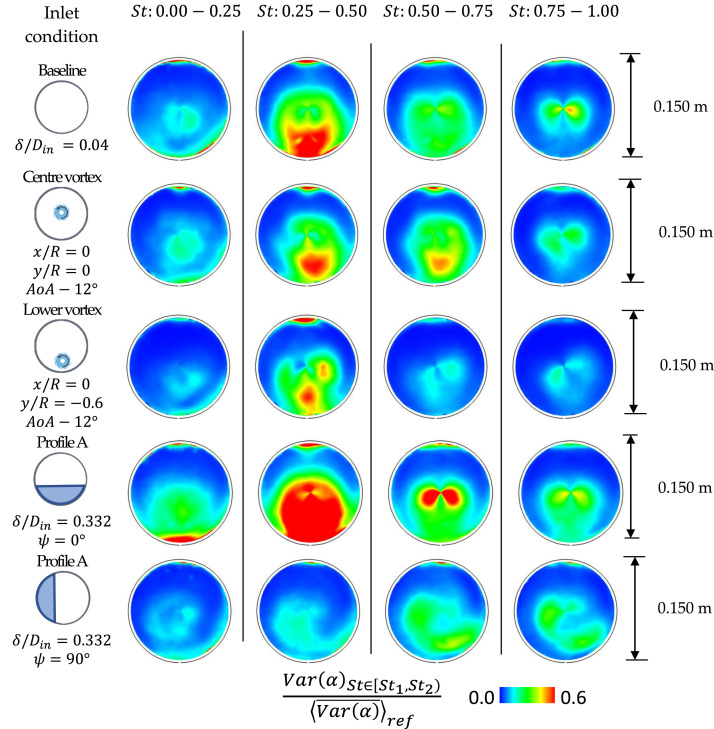
Spectral signature of ΔSt = 0.25 of the AIP swirl angle fluctuations for baseline inlet conditions, inlet vortex and inlet Profile A configurations.

The spectral analysis of the AIP flow field reveals the main frequency content of the flow distortion (Figure 11) with a resolution of ΔSt = 0.2. The main fluctuations of the swirl angle for the baseline case (Figure 11) are contained with a Strouhal number band between St = 0.25–0.50. Gil-Prieto et al.^36^ demonstrated that these frequencies are linked with the first switching mode (FSM, St = 0.42). This unsteady mode is dominated by the alternation between bulk swirl (SD = ±1) to paired swirl (SD = 0) patterns. On the other hand, the higher frequencies are linked with the first vertical mode^36^ (FVM, St = 0.5–1.0).

Besides the interest in baseline configurations and inlet conditions, there is a need to understand if changes in the inlet condition can modify the underpinning frequency of the flow distortion. The introduction of the vortex does not change the main frequency contribution of the flow distortion (Figure 11), which remains in a band St = 0.25–0.50 independently from the vortex strength and for the different vortex ingestion positions. In case of vortex ingestion at the inlet centre (x/R = 0, y/R = 0, AoA = −12°), the distribution and the strength of the fluctuations across the different bands remain constant relative to the inlet baseline case with no vortex ingestion (Figure 11). A similar behaviour was also observed when the vortex was ingested towards the left side of the inlet (x/R = −0.6, y/R = 0, AoA = −12°). Instead, for the vortex ingestion at the lower side of the inlet (x/R = 0, y/R = 0, AoA = −12°), there was a noticeable reduction of the strength of the fluctuations across the frequency bands, especially between St = 0.5–1.0 (Figure 11). Since the strength of high-frequency fluctuations which may promote propulsion system instability is suppressed, this highlights the potential benefit of the vortex ingestion at the lower inlet side.

On the other hand, the impact of the inlet total pressure profile is much more pronounced. Relative to the datum inlet condition (δ/D_in_ = 0.04), Profile A (δ/D_in_ = 0.332, ψ = 0°) promotes the swirl fluctuations at St = 0.0–0.25 (Figure 11). Conversely, increasing ψ from 0° to 90° causes mainly an increase of the frequency of the swirl fluctuations St = 0.25–0.50 to St = 0.5–0.75 (Figure 11). These effects are consistent with those identified by McLelland et al.^52^ based on the unsteadiness of the velocity magnitude. It is envisaged that the decrease of momentum of the lower inlet side due to the thick inlet profile interacts with the flow on the lower side of the S-duct and reduces the momentum in the lower region of the AIP. Consequently, this produces wider oscillations of the swirl angle (Figure 8(e)) with reference to the baseline inlet conditions (Figure 4(b)). Since larger fluctuations are associated with a larger oscillation period, the frequency of the swirl angle fluctuations reduces (Figure 11).

Overall, it can be concluded that inlet vortices have an impact on the strength of the swirl angle fluctuations, but they do not change the main spectral signature. On the other hand, inlet total pressure profiles had a greater impact and they modified both the amplitude and the frequency of the swirl angle fluctuations at the AIP and may impose a more challenging operating condition for the propulsion system.

### EVT predictions for peak swirl distortion

Even though average distortion levels are of interest, the assessment of the peak distortion levels is the vital aspect of the intake-engine compatibility studies.^37^ Although the peak distortion may be observed during experimental testing, due to its random nature, it can appear for observations beyond the experimental testing time. Previous work demonstrated that peak distortion tends to increase with data acquisition time and estimated the required testing time to observe the peak distortion with extreme value theory (EVT).^35,65^ However, often aero-engine tests result in time-consuming and expensive experimental campaigns, and therefore, it is important to have methods that can estimate the likely critical peak distortion levels based on the measured datasets. An overview of the current state of the art on distortion synthesis and estimation techniques and relative accuracy is included in the current standard SAE AIR 5826B,^71^ which highlights how maximum value statistics should be applied to ensure (1) that the near-maximum level of distortion has been measured during experimental tests and (2) that the estimated peak distortion is near the maximum values most likely to be experienced during the aircraft lifetime. EVT is a potential method to address this issue. In this study, the area-averaged swirl intensity $\bar{SI}$ at each TR-PIV timestep was used to build the EVT model and the central consideration is to determine if the EVT approach can be successfully used for this type of TR-PIV swirl data and to highlight the impact of the inlet conditions on the relationship between the measured peak distortion and the expected maximum. The EVT model parameters were evaluated separately for each dataset. The threshold for the EVT model has been chosen based on the value that minimizes the RMS fit error of the log-likelihood function (equation (4)) for modelling the exceedances $Y_{i}$ (equation (2)). It is highlighted that the peak-over-threshold is one of the possible formulations of the EVT model. Alternative formulations are discussed by Coles.^66^ The analysis of the sensitivity of the EVT model to the threshold selection was carried out on representative configurations including the uniform inlet, the ingestion of the vortex at the inlet centre (x/R = 0, y/R = 0, AoA = −12°) and with Profile A (δ/D_in_ = 0.332,  ψ=0°). An increase of the threshold by +1 deg produced an increase of 0.51°, 0.31° and 0.01° for the predicted peak $\;\bar{SI}$ and an increase of 0.82°, 0.61° and 0.19° for the confidence interval CI (equation (13)), respectively, for the different inlet configurations. These variations are an order of magnitude lower compared with extreme values predicted by the model. A summary of the model parameters is reported in Table 2.

**Table 2.** EVT model parameters for the different test cases.

	Baseline inlet conditions	x/R = 0 y/R = 0 AoA = −12°	x/R = −0.6 y/R = 0 AoA = −12°	x/R = 0 y/R = −0.6 AoA = −12°	$\delta /D_{\mathrm{in}}$ 0.332 ψ=0°	$\delta /D_{\mathrm{in}}$ 0.332 ψ=90°	$\delta /D_{\mathrm{in}}$ 0.572 ψ=0°	$\delta /D_{\mathrm{in}}$ 0.572 ψ=90°
ξ	−0.127	−0.122	−0.171	−0.225	−0.150	−0.196	−0.136	−0.154
σ	1.018	1.006	1.424	1.587	1.387	0.846	1.225	0.663
k	876	958	2618	4646	3005	2692	1427	1785
μ(°)	10.4	10.9	9.6	6.5	12.2	11.3	14.0	13.2

**Figure 12.**
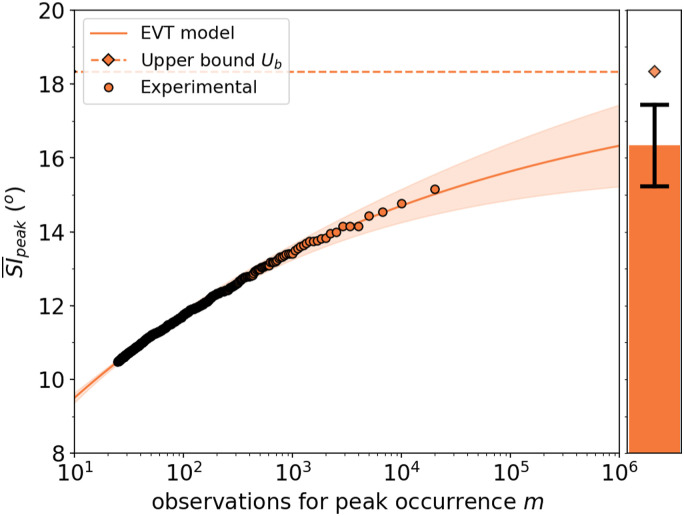
EVT predictions for the peak $\bar{\mathrm{SI}}$ at the AIP baseline inlet conditions (continuous line) and projected upper bound (dashed line) based on peak $\bar{\mathrm{SI}}$ experimental observations (dotted line).

The EVT model was used to estimate the peak area-averaged swirl intensity (SI) for a number of observations two orders of magnitude greater than the experimental ones, which is a typical practice for these statistical assessments^65^ (Figure 12). The application of the EVT also estimated the asymptotic level $U_{b}$ and relative uncertainty for the peak $\bar{SI}$ for an infinite number of observations (Figure 12). For baseline inlet conditions (δ/D_in_ = 0.04, no inlet vortex), the measured peak $\bar{SI}$ was 15.2° (based on 2 × 10^4^ observations) and the EVT model predicted a peak $\bar{SI}$ of 16.3 ± 1.1° and an upper bound of 18.4 ± 0.1° (Figure 12). Thus, relative to the measured datasets, the predicted peak $\bar{SI}$ could be up to 3° greater. Although the difference between observed and predicted peak $\bar{SI}$ difference seems small, previous work showed that an increase of 5° of SI could produce a loss of up to approximately 5% in the compressor pressure ratio,^47^ and it could reduce the stability range of a transonic axial compressor by up to 5%.^72^ Thus, because of this potential impact on the compressor stability, the expected higher peak $\bar{SI}$ is a key consideration.

In this experimental study, it was possible to construct an EVT model and to extract peak $\bar{SI}$ values from a population of 2 × 10^4^ individuals, which corresponds to 5s of testing time with an acquisition frequency of 4 kHz. The EVT model estimated the peak $\bar{SI}$ values for a projection of one million samples which corresponds to 250s of testing time. Thus, the EVT model can be a useful tool to estimate the peak events for very long observation times in those setups where prolonged experimental observations would be prohibitive because of the high running costs.

**Figure 13.**
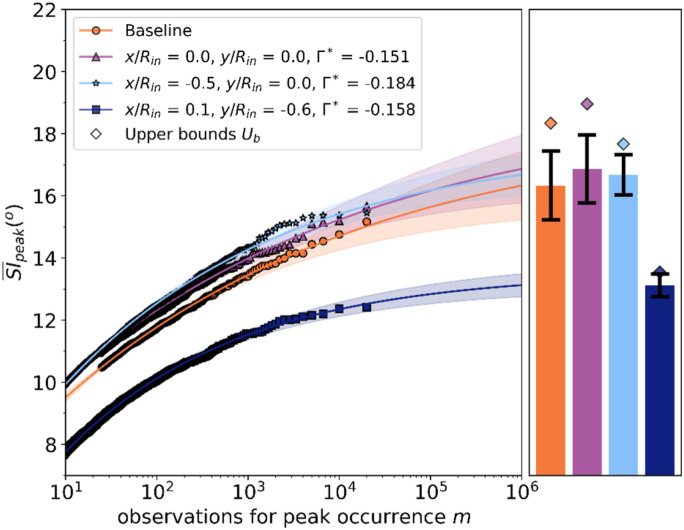
EVT predictions for the peak $\bar{\mathrm{SI}}$ at the AIP baseline inlet conditions and inlet vortex ingestion configurations.

The EVT method has been applied to the cases of non-uniform inlet conditions. The impact of the vortex onto the peak $\bar{SI}$ depended on the vortex ingestion position at the inlet (Figure 13). As demonstrated in previous sections, the ingestion of the vortex at the centre (x/R = 0, y/R = 0, AoA = −12°) or left side (x/R = −0.6, y/R = 0, AoA = −12°) of the inlet caused a very small increase of time-averaged swirl angle and peak swirl intensity. For these configurations, the measured peak $\;\bar{SI}$ among 2 × 10^4^ observations was about 0.7° higher compared to the baseline inlet conditions (Table 3). The EVT also indicated that for 10^6^ observations and in the case of centre and left inlet vortex, the peak $\bar{SI}$ is up to 1.7° higher than the uniform inlet configuration (Figure 13). For these 3 configurations (baseline, centre and left inlet vortex), the shape parameter ξ and the threshold μ  of the model were comparable (Table 2). As a consequence, the estimated rate of increase of the peak $\;\bar{SI}$ between 10^4^ and 10^6^ observations, up to 2.0°, was similar across these configurations. It can be argued that inlet vortices at the centre and left side positions increase peak swirl distortion levels at the AIP of a similar amount. On the other hand, the measured peak $\bar{SI}$ for the ingestion of the vortex at the lower inlet side (x/R = 0, y/R = −0.6, AoA = −12°) was 1.5° lower than for inlet baseline conditions (Figure 13) which is consistent with the time-averaged swirl distribution (Figure 6) and tip peak swirl intensity (Figure 7). The EVT estimate of peak $\bar{SI}$ increases slightly from 12.5° to 13.1° for an increase in observations from 10^4^ to 10^6^ (Table 3), which is a much lower increase than other configurations. Indeed, the shape factor ξ and the threshold μ of the fitted EVT model are much lower than the other vortex configurations (Table 2), and therefore, the growth rate of the peak distortion diminished.

**Table 3.** Prediction of the peak $\bar{\mathrm{SI}}$ for 10^6^ observations and experimental peak $\bar{\mathrm{SI}}$ for baseline inlet conditions and inlet vortex ingestion configurations.

	*Baseline*	*x/R = 0 y/R = 0 AoA = −12°*	*x/R = −0.6 y/R = 0 AoA = −12°*	*x/R = 0 y/R = −0.6 AoA = −12°*
${\bar{SI}}_{peak,\;\;exp}\left({}^{\circ}\right)$	15.2	15.7	15.5	12.4
${\bar{SI}}_{m=10^{6},\;\;EVT}\left({}^{\circ}\right)$	16.3 ± 1.1	16.9 ± 1.1	16.8 ± 0.7	13.2 ± 0.4
$U_{b,\;\;EVT}\left({}^{\circ}\right)$	18.4	19.1	17.9	13.6

**Figure 14.**
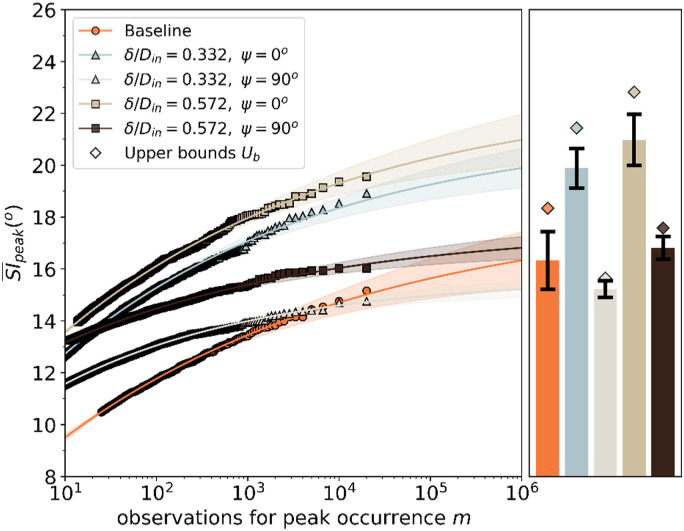
EVT predictions of the peak $\bar{\mathrm{SI}}$ at the AIP for baseline inlet conditions and inlet total pressure profiles configuration.

The imposition of inlet total pressure profiles at the S-duct inlet had, in general, a much higher impact on the time-averaged and unsteady swirl distortion at the AIP (Section III.C and D). The measured peak $\bar{SI}$ increased from 15.2° to 18.9° and 19.6° when the inlet total pressure profile was increased from baseline (δ/D_in_ = 0.04) to Profile A (δ/D_in_ = 0.332) and to Profile B (δ/D_in_ = 0.572), respectively, for 2 × 10^4^ observations (Table 4). The EVT model predicted a further increase of the peak $\bar{SI}$ by up to 1.8° and 2.4°, respectively, for 10^6^ observations (Table 4). On the other hand, when the profiles were oriented at ψ=90°, the predicted peak $\bar{SI}$ was comparable with the baseline configuration (Figure 14). In these configurations, the EVT predicted a peak $\bar{SI}$ up to 15.5° and 17.2° for Profile A and Profile B, respectively, for 10^6^ observations.

**Table 4.** Prediction of the peak $\bar{\mathrm{SI}}$ for 10^6^ observations and confidence intervals for baseline inlet conditions and inlet total pressure profiles configurations.

	Baseline	$\delta /D_{\mathrm{in}}$ 0.332 ψ=0°	$\delta /D_{\mathrm{in}}$ 0.332 ψ=90°	$\delta /D_{\mathrm{in}}$ 0.572 ψ=0°	$\delta /D_{\mathrm{in}}$ 0.572 ψ=90°
${\bar{SI}}_{peak,\;\;exp}\left({}^{\circ}\right)$	15.2	18.9	14.8	19.6	16.0
$SI_{m=10^{6}}\left({}^{\circ}\right)$	16.3 ± 1.1	19.9 ± 0.8	15.2 ± 0.3	21.0 ± 1.0	16.8 ± 0.4
$U_{b,\;\;EVT}\left({}^{\circ}\right)$	18.4	21.4	15.6	23.0	17.5

**Figure 15.**
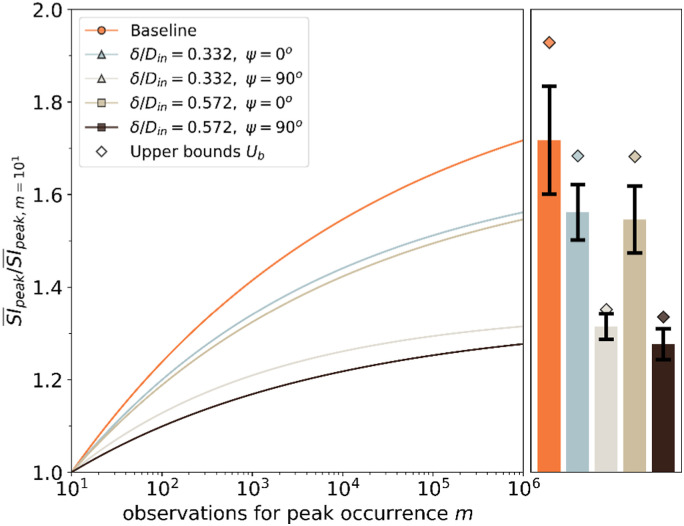
EVT model growth rate of the peak $\bar{\mathrm{SI}}$ at the AIP for baseline inlet conditions and inlet total pressure profiles configuration.

The orientation of the inlet total pressure profiles at ψ=0° exhibited a much higher rate of growth of the estimated peak SI compared with the ones at ψ=90°. This is more evident if the peak $\bar{SI}$ is normalized against a reference peak $\bar{SI}$ for m = 10 observations (Figure 15). The baseline inlet configuration (δ/D_in_ = 0.04) exhibited a higher growth rate with a normalized peak $\bar{SI}$ of 1.6. The growth ratio of the inlet profiles A and B oriented at ψ=0° was slightly lower than the baseline configuration. On the other hand, the growth ratio reduced notably when the inlet profiles A and B were oriented at ψ=90° (Figure 15). It is believed that the growth rate of the peak distortion is related to the flow unsteadiness at the AIP. The area-averaged unsteady swirl angle reduced from 12° to 9° approximately when the inlet profiles were rotated from 0° to 90°. Thus, it is posited that although the EVT model is required to estimate the peak distortion, the swirl angle unsteadiness is a key indicator and highlights the need for synchronous field measurements with sufficient spatial resolution.

The EVT also provides an estimate for the projected upper bound which highlights the differences in the EVT signatures for the different configurations. The growth rates for the baseline and Profile A (δ/D_in_ = 0.332,  ψ=90°) configuration are notably different. For example, the measured peak $\bar{SI}$ at 1 × 10^4^ observations is the same (14.2°) for both configurations, and for the full measured dataset of 2 × 10^4^, the peak $\bar{SI}$ is 15.2° for the baseline and slightly lower at 14.8° for the Profile A (ψ=90°) (Figure 14). However, as the EVT model reveals that the growth rate of the extreme events is notably different for these two configurations and the projected upper bound $U_{b}$ for the baseline inlet configuration is 18.4° compared with 15.6° for the inlet Profile A (δ/D_in_ = 0.332,  ψ=90°). Thus, this work demonstrates that the evaluation of the distortion based only on the measured peak distortion may be misleading. Instead, it is recommended to evaluate the underlying unsteadiness and the rate of growth of the extreme values with a statistical model such as EVT to evaluate the likely distortion presented to the engine.

In conclusion, both inlet vortex and inlet total pressure profiles increased the levels of peak distortion at the AIP. Overall, maximum levels of distortion were observed for inlet total pressure profiles oriented at ψ=0°, while for ψ=90°, the peak distortion was similar to uniform inlet conditions. Inlet vortices at the centre and left side of the inlet section caused an increase in peak distortion which was comparatively lower than inlet total pressure profiles, while the ingestion of inlet vortices at the lower side of the inlet caused a notable reduction of the peak distortion levels at the AIP.

## Conclusions

This work assessed the impact of the inlet conditions on the unsteady aerodynamics of complex intakes. While most of the previous work on S-duct intakes focused on canonical assessments of the flow distortion with uniform inlet conditions, this work represents a notable step forward in the characterization of the unsteady distortion for various inlet conditions. This investigation assessed the impact of inlet vortices of different strengths and positions and inlet total pressure profiles’ thickness and orientation.

The presence of an inlet vortex influenced the secondary flows of the intake and augmented the vortex which was spinning in the same direction of the inlet vortex. This disrupted the characteristic S-duct switching mode and biased the swirl pattern towards bulk swirl events. In general, the inlet vortex had little or no impact on the swirl angle unsteadiness and on the peak swirl intensity. However, when the vortex was ingested at the inlet lower side, the swirl unsteadiness reduced by 25% compared to other inlet vortex ingestion positions and a reduction of the unsteadiness was observed across all the frequency bands. For this configuration, the recorded peak swirl intensity reduced by 3° compared to the uniform inlet configuration.

Inlet total pressure profiles had a strong impact on the AIP flow distortion. In the baseline symmetric orientation, they increased the swirl angle unsteadiness and the peak swirl intensity. The azimuthal rotation of the inlet profile caused a bias of the secondary flows and the growth of one of the Dean vortices. This effect was similar but more augmented to the effect of inlet vortices. The azimuthal rotation caused a raise in swirl angles; however, it reduced the unsteadiness because the swirl bias induced a more stable condition for the AIP flow. The frequency of the swirl fluctuations depended strongly on azimuthal rotation of the inlet profile. For the symmetric inlet condition, the frequency fluctuations reduced. However, for the asymmetric inlet condition, they increased to a range of St = 0.5–1.0. This may represent an important condition for the operability of the propulsion system. Overall, this work shows that the characteristics of the S-duct flow distortion depend on the inlet conditions, and thus, it is recommendable to evaluate the intake duct flow distortion also for a range of inlet conditions other than uniform.

The extreme value theory estimated the peak distortion beyond the experimental observations. For some configurations, the EVT predicted an increase in swirl intensity by up to 3° compared to the experimental values. Thus, this projected increase of swirl intensity should be considered for the design of distortion-tolerant propulsion systems. The EVT revealed that the flow unsteadiness may have a large impact on the projected upper bound and growth rate of the extreme events. The growth rate of the extreme events may be substantially different also in configurations in which the same peak distortion level was measured experimentally. Indeed, it is demonstrated that the evaluation of distortion based on the measured peak distortion can be misleading, and it is recommended to use a statistical model such as EVT to evaluate the growth rate and projected peak distortion levels. This evidences also the need of unsteady measurements of the flow distortion with a high spatial resolution.

## Appendix

### Nomenclature

CI: = confidence interval for the EVT model
EVT: = extreme value theory
Re: = Reynolds number
SAE: = Society of Automotive Engineers
SD: = Swirl Directivity (SAE)
SI: = swirl intensity (SAE), °
SP: = swirl pairs (SAE)
*St*: = Strouhal number, $f_{s}D/v$
UHBR: = ultra-high bypass ratio
x: = horizontal coordinate
y: = vertical coordinate
A: = area, m^2^
AIP: = aerodynamic interface plane
AoA: = angle of attack, °
c: = chord, mm
cov: = covariance
$D_{in}$: = diameter at the inlet of the S-duct, mm
$D_{out}$: = diameter at the outlet of the S-duct, mm
$f_{s}$: = sampling frequency, Hz
H: = vertical offset of the S-duct, m
k: = number of exceedances
L: = length of the S-duct, m
M: = Mach number
PDF: = probability density function
r: = radial coordinate, m
std: = standard deviation
$U_{b}$: = upper bound of the EVT model
v: = velocity component, m/s
var: = variance
z: = streamwise coordinate
α: = absolute swirl angle, °, $\tan^{-1}\left(v_{\theta }/v_{z}\right)$
Γ: = vortex circulation, m/s^2^
δ: *=* boundary layer thickness, mm
μ: = exceedances threshold, °
ξ: = shape parameter of the EVT model
θ: = tangential coordinate, °
σ: = scale parameter of the EVT model
ψ: = azimuthal angle of orientation of the inlet total pressure profile, °
〈 〉: = time-average
$\bar{\;\;}$: = area-average
